# Reprogramming Chromosome Ends by Functional Histone Acetylation

**DOI:** 10.3390/ijms25073898

**Published:** 2024-03-31

**Authors:** W. Alex Meltzer, Aditi Gupta, Phyo Nay Lin, Robert A. Brown, Daniel S. Benyamien-Roufaeil, Raju Khatri, Anup A. Mahurkar, Yang Song, Rodney J. Taylor, Michal Zalzman

**Affiliations:** 1 Department of Biochemistry and Molecular Biology, University of Maryland School of Medicine, Baltimore, MD 21201, USAaditi.gupta@nih.gov (A.G.); phyonaylinn@gmail.com (P.N.L.); rabrown@som.umaryland.edu (R.A.B.); dbenyamienroufaeil@som.umaryland.edu (D.S.B.-R.); ghiraju@gmail.com (R.K.); 2 Institute for Genome Sciences, University of Maryland School of Medicine, Baltimore, MD 21201, USA; amahurkar@som.umaryland.edu (A.A.M.); ysong@som.umaryland.edu (Y.S.); 3 Department of Otorhinolaryngology-Head and Neck Surgery, University of Maryland School of Medicine, Baltimore, MD 21201, USA; rtaylor@som.umaryland.edu; 4 Marlene and Stewart Greenbaum Cancer Center, University of Maryland School of Medicine, Baltimore, MD 21201, USA; 5 The Center for Stem Cell Biology and Regenerative Medicine, University of Maryland School of Medicine, Baltimore, MD 21201, USA

**Keywords:** telomeres, ZSCAN4, epigenetics, histone acetylation, pluripotency, stem cells, cancer, CRISPR, over-expression, next-gen sequencing

## Abstract

Cancers harness embryonic programs to evade aging and promote survival. Normally, sequences at chromosome ends called telomeres shorten with cell division, serving as a countdown clock to limit cell replication. Therefore, a crucial aspect of cancerous transformation is avoiding replicative aging by activation of telomere repair programs. Mouse embryonic stem cells (mESCs) activate a transient expression of the gene *Zscan4*, which correlates with chromatin de-condensation and telomere extension. Head and neck squamous cell carcinoma (HNSCC) cancers reactivate ZSCAN4, which in turn regulates the phenotype of cancer stem cells (CSCs). Our study reveals a new role for human ZSCAN4 in facilitating functional histone H3 acetylation at telomere chromatin. Next-generation sequencing indicates ZSCAN4 enrichment at telomere chromatin. These changes correlate with ZSCAN4-induced histone H3 acetylation and telomere elongation, while CRISPR/Cas9 knockout of ZSCAN4 leads to reduced H3 acetylation and telomere shortening. Our study elucidates the intricate involvement of ZSCAN4 and its significant contribution to telomere chromatin remodeling. These findings suggest that ZSCAN4 induction serves as a novel link between ‘stemness’ and telomere maintenance. Targeting ZSCAN4 may offer new therapeutic approaches to effectively limit or enhance the replicative lifespan of stem cells and cancer cells.

## 1. Introduction

Telomeres are repetitive sequences at the ends of all chromosomes that shorten with every cell division. As such, telomere shortening functions as a biological “clock” that limits cells’ ability to replicate indefinitely [1,2,3,4,5,6]. As long as this countdown timer is not reset, normal adult replicating cells will cease to divide within approximately 50 cell divisions. Conversely, highly replicative immortal cells, such as pluripotent stem cells and cancer cells, require chromatin-modifying factors to permit a regulated accessibility of the telomere DNA. This enables telomere maintenance mechanisms to repair and extend the telomeres. Although factors involved in telomere chromatin condensation have been identified [7], the mechanisms controlling the switch to a more accessible chromatin are not well understood.

The murine *mZscan4* is an early embryonic development gene, and a key factor for maintaining embryonic stem cells (ESCs) [8] and germ cells [9]. Furthermore, it plays a critical role in maintaining genome stability of early-stage embryos and pluripotent stem cells such as ESCs and induced pluripotent stem cells (iPSCs) [8,10]. Importantly, ZSCAN4 promotes telomere maintenance and stability by inducing the DNA homologous recombination (HR)-based alternative lengthening of telomeres (ALT) pathway in embryos and iPSCs [8,11,12,13,14].

The involvement of the murine *Zscan4* (*mZscan4*) in chromatin remodeling has been suggested because a transient expression of *mZscan4,* correlates with global DNA demethylation and chromatin de-condensation [8,12,15,16]. Further studies have demonstrated that mZSCAN4 facilitates nuclear reprogramming during the generation of iPSCs [9,15,17]. It was also shown to be involved in the preservation of developmental potency of mouse embryonic stem (mES) cells [18,19,20]. Conversely, the activity of the human ZSCAN4 protein remains unknown. However, it has been suggested to have significance in telomere stability, as it interacts with the telomeric shelterin complex [21,22], and its gene expression is upregulated by genotoxic stress [19,23] and telomere attrition [11]. Although the human ZSCAN4 is not expressed in adult differentiated cells, it is re-expressed in various types of cancer [22,24]. We recently reported that the human ZSCAN4 marks and regulates the cancer stem cell (CSCs) phenotype in head-and-neck squamous-cell carcinoma (HNSCC) by altering the epigenetic profile at the promoters of CSC factors [24]. In this study, we show for the first time a novel role of human ZSCAN4 in facilitating histone H3 acetylation at the telomere chromatin and determine its significant contribution to telomere maintenance in cancer stem cells.

## 2. Results

Telomere shortening restricts cell proliferation and ultimately leads to aging and loss of cell function [25]. ZSCAN4 was shown to play a role in telomere maintenance of mouse embryonic stem (mES) cells [8,15,17]. Moreover, an ectopic expression of the human *ZSCAN4* in mouse embryonic fibroblasts leads to higher efficiency in generation of induced pluripotent stem cells (iPSC) [17]. ZSCAN4 was further shown to indirectly interact with the telomeric shelterin complex [21,22]. However, the function by which ZSCAN4 protein acts on the telomeric DNA or chromatin still remained unknown.

### 2.1. Human ZSCAN4 Co-Localizes with the Telomere Region

**Figure 1:**
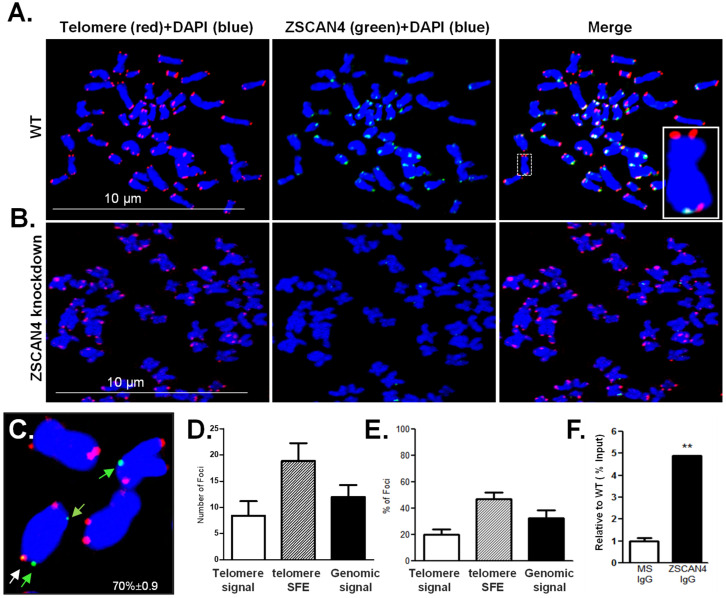
ZSCAN4 forms foci with preference to the telomere region. (**A**) Representative images of co-localization of telomeres (red, Cy3-PNA probe) and anti-ZSCAN4 (green), in metaphase spreads (Tu167 WT cells). Chromosomes stained with DAPI (blue). (**B**) ZSCAN4 knockdown cells used as negative controls. (**C**) Representative image showing chromosomes with arrows pointing at colocalization of ZSCAN4 with telomeres (white arrow) and telomere signal-free ends (SFE) (green arrows). (**D**) Quantification by ImageJ (Fiji/ImageJ 2.14) of ZSCAN4 with chromosome ends by confocal analyses. (**E**) The percentage of foci revealed a preference for telomere signal-free ends (SFE) (*p* ≤ 0.001). Error bars indicate S.E.M. Data are based on three independent experiments. (**F**) Telomere ChIP assays using anti-ZSCAN4 IgG indicate interaction with the telomeres. Values are relative to % input shown as mean ± S.E.M. from three independent experiments; Control used: normal IgG. Asterisks indicate ** *p* ≤ 0.01.

The mouse mZSCAN4 was previously shown to be enriched at the telomeres [8]. To assess the localization of human ZSCAN4 protein, we performed human ZSCAN4 immunostaining, along with telomere fluorescence in situ hybridization (T-FISH) assays, followed by confocal microscopy in Tu167 cell lines (Figure 1A). ZSCAN4 knockdown cells were used as negative controls (Figure 1B). T-FISH analyses indicated colocalization of ZSCAN4 with telomere and “signal-free ends” (SFE) (Figure 1C). However, the corresponding chromosomes of SFEs showed no end-to-end fusions. Therefore, we infer that these shortened ends retain some end-protection, and the signals are likely below the detection threshold. Quantification of the colocalization of ZSCAN4 with the telomeres further showed its positive signals with telomeres and with telomere SFEs (Figure 1D,E). Our results reveal that 70 ± 0.9% (mean ± S.E.M) of ZSCAN4 foci are associated with the telomere and telomere SFEs (*p* ≤ 0.01) (Figure 1D,E). ZSCAN4 chromatin immunoprecipitation (ChIP) assays followed by telomere qPCR further validated a significant enrichment of ZSCAN4 protein at the telomeres (*p* ≤ 0.005) (Figure 1F), as compared to IgG control (negative), and anti-RNA PolII (positive). RPLP0 was used to demonstrate equal loading. Our results indicate that ZSCAN4 protein is significantly enriched in the telomere region.

While the human ZSCAN4 is not expressed in adult differentiated cells, it is re-expressed in various types of cancer [22,24]. A recent report indicated that the human ZSCAN4 marks and regulates the cancer stem cell (CSCs) phenotype in head-and-neck squamous-cell carcinoma (HNSCC) by altering the epigenetic profile at the promoters of CSCs factors [24]. This study demonstrates for the first time a novel role of human ZSCAN4 in promoting histone H3 acetylation at the telomere chromatin and its significant contribution to telomere maintenance in cancer stem cells. Understanding how ZSCAN4 modulates the telomeric chromatin is crucial for developing new therapeutic approaches to target cancer stem-cell replicative lifespan.

### 2.2. Human ZSCAN4 Facilitates Telomere Extension

We have previously shown that ZSCAN4 is transiently expressed in human squamous cell carcinoma cells and increases the frequency of cancer stem cells [24]. Therefore, the effect of human ZSCAN4 on telomeres was assessed in telomerase-positive (Tu167) and -negative (U2OS) cells using a doxycycline-inducible tet-ZSCAN4 lentiviral vector [8,15,16,26]. First, to validate telomerase status in our cell lines, we assessed the expression of human telomerase reverse transcriptase (TERT), the enzyme subunit of telomerase (Supplementary Figure S1A) and TERC, the RNA component of telomerase (Supplementary Figure S1B) by qRT-PCR and telomerase activity assays (Supplementary Figure S1C). HeLa, 012SCC, SKBr3, and SW480 cells were used as telomerase-positive controls. As expected, the results confirmed that Tu167 cells are telomerase-positive, while U2OS are telomerase-negative (Supplementary Figure S1A–C).

**Figure 2:**
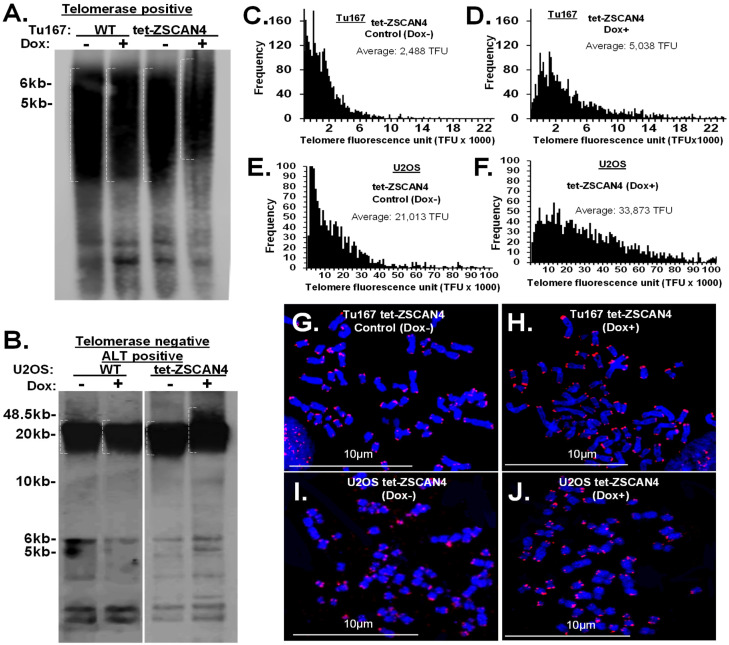
Transient induction of ZSCAN4 modulates telomere length. (**A**) Southern blot analyses demonstrate Dox induction of ZSCAN4 for 48 h in telomerase-positive tet-ZSCAN4 Tu167 cells, and (**B**) in telomerase-negative tet-ZSCAN4 U2O-S cells, this leads to telomere extension at 72 h compared to untreated (Dox−) isogenic cells. Dox-treated (Dox+) and untreated (Dox−) wild-type cells were used as additional controls. (**C**) Q-FISH analyses show a distribution diagram of relative telomere length in non-induced Tu167 cells (Dox-) and (**D**) ZSCAN4 induced cells (48 h Dox+). (**E**) U2OS cells (Dox−) compared to (**F**) ZSCAN4 induced (Dox+). (**G**–**J**) Representative images of telomere FISH in metaphase spreads. Telomeres were detected using an Alexa546-DNA probe (red). Chromosomes stained with DAPI (blue). Q-FISH data were analyzed using TFL-Telo software (results of pooled nuclei, totaling > 2500 telomeres). The data were reproduced in three independent experiments.

Next, to determine the effect of human ZSCAN4 on telomeres in telomerase-positive (Tu167) and -negative (U2OS) cells, we used a doxycycline-inducible tet-ZSCAN4 lentiviral vector, as previously described [24,27]. We then generated cell lines in which ZSCAN4 is induced by addition of doxycycline (Dox), a tetracycline analog, to the culture medium. We then performed telomere Southern blot analyses using control cells without (Dox-) or induction of ZSCAN4 by doxycycline (Dox+) in both cell lines (Tu167 and U2OS) for 48 h. Isogenic wild-type (WT) cells treated with Dox and untreated cells were used as additional controls to exclude the effect of doxycycline. As expected, Dox treatment of WT control cells had no significant effect on telomere length (Figure 2A,B). Conversely, Southern blot analysis indicated that ZSCAN4 was induced by Dox for 48 h in Tu167 cells (Figure 2A), and U2OS cells (Figure 2B) led to an increase in telomere length.

To validate these data, we also performed quantitative-telomere fluorescence-in-situ hybridization (Q-FISH) [8,28] analyses in tet-ZSCAN4 cells (Tu167 and U2OS) with and without transient ZSCAN4 induction. Our data indicate a shift up of the shorter telomere signal intensities, leading to an overall 2-fold increase in the average telomere fluorescence units (TFUs) after ZSCAN4 induction in Tu167 cells (Figure 2C,D), and a 1.6-fold increase in U2OS compared to the uninduced controls (Figure 2E,F). Representative corresponding images (Figure 2G–J). These results suggest that human ZSCAN4 interacts with the telomeres to promote telomere editing irrespective of telomerase status.

### 2.3. Telomere-Specific Next-Gen ChIP-Seq Reveals Significant Enrichment of ZSCAN4

**Figure 3:**
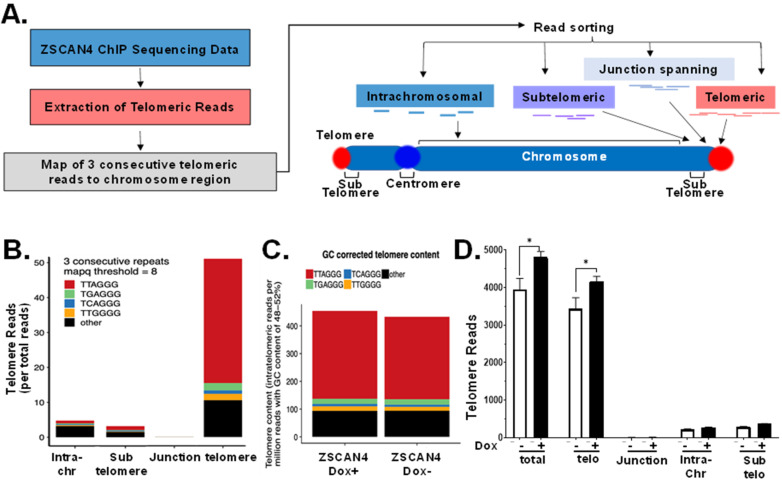
ChIP-next-generation sequencing analyses of Tu167 (telomerase + cells) revealed enrichment of ZSCAN4 in telomere sequences. (**A**) Schematic illustration of ZSCAN4 ChIP-Seq analyses by TelomereHunter and sequence classification of telomere repeats by location and proximity to telomeres. (**B**) ZSCAN4 ChIP-Seq followed by TelomerHunter analysis showing enrichment of ZSCAN4 at telomere repeats compared to other NNNGGG sequences. (**C**) ZSCAN4 Dox induction (Dox+) in Tu167 led to a further increase in ZSCAN4 at telomere repeats compared with the control (Dox−). (**D**) Further analyses showed that telomere-specific repeats were enriched at the ends of the chromosomes but not at other sites after ZSCAN4 induction (Dox+). * *p* < 0.05.

Previous studies using GFP-ZSCAN4 chromatin immunoprecipitation (ChIP) followed by next-generation sequencing have indicated that Zscan4 is a microsatellite binding factor that protects fragile genomic regions from DNA damage during embryogenesis [29,30]. Since our studies found a significant colocalization of ZSCAN4 antibodies with the telomeres, here, we investigated its interaction with G-rich repeat sequences by chromatin immunoprecipitation (ChIP), followed by next-gen ChIP-seq. As telomeres are non-coding DNA repeats, they are eliminated in the first discovery phase of most next-generation sequencing analysis tools. This is because such tools were developed to search for peaks and motifs for gene discovery and are, therefore, designed to ignore repeat sequences. Thus, to validate the potential enrichment of ZSCAN4 in telomeres, we used TelomereHunter [31]. This ChIP-seq analysis tool is designed to search within the raw sequencing data, for predefined telomere variant repeats of the NNNGGG type (and the reverse complement), where ‘N’ can stand for A, C, G or T. It further allows us to assess if the location of the repeat is within the telomeres, the subtelomeres, or otherwise in other chromosome regions [32] (Figure 3A).

To calculate the telomeric reads per GC content-matched million reads, the number of telomeric reads was normalized to the number of reads with comparable GC content (48–52%) and multiplied by 10. Next, the reads were classified based on their sequence location relative to the telomeres. ChIP-seq data indicated a significant enrichment of ZSCAN4 in telomere repeats compared to other NNNGGG sequences (Figure 3B). As additional internal controls, we used Alu repeats [25] using RepeatMasker [33,34]. Our data indicated that ZSCAN4 induction leads to enrichment in its binding to the telomere sequence TTAGGG repeats compared to non-telomeric repeats (Figure 3C). Further analyses of ZSCAN4 ChIP-seq showed that the increase in these repeats was indeed located at the telomeres, at the end of the chromosomes, but not in intra-chromosomal regions (Figure 3D).

### 2.4. ZSCAN4 Facilitates H3 Histone Acetylation at the Telomere Region

We recently reported that human ZSCAN4 facilitates histone H3 acetylation at the promoters of the core pluripotency factors OCT3/4 and NANOG [24]. When histones are acetylated, DNA becomes loosely coiled around histones. Here, we sought to assess the effect of ZSCAN4 on histone H3 acetylation, specifically at telomeres, by chromatin immunoprecipitation (ChIP) using validated antibodies for histone 3 lysine 14 acetylation (H3K14ac) and lysine 18 acetylation (H3K18ac). Our dot blot data confirmed an increase in the telomere signal after ZSCAN4 induction (Dox+) (Supplementary Figure S2A). Therefore, to account for telomere extension and detect an increase in histone acetylation, Dox+ samples and their inputs were diluted 1.5-fold. We then performed a dot blot analysis using telomere probes. Our ChIP-dot blots showed an increase in H3K14ac and H3K18ac on telomere chromatin (Supplementary Figure S2B). Input samples (10%) were used as loading controls, and normal rabbit IgG was used as negative control for all the probes. To compensate for telomere extension, we performed dot blot analysis with sample inputs (*n* = 3 per condition). We also used mutated telomere probe (Telo-Mut) (Supplementary Figure S2C), which showed no significant histone H3 acetylation at the telomeres, whereas input was detectable only after an extended exposure time. As additional controls, we used Alu probes, as Alu are 300-base-pair long, GC-rich repetitive transposable elements dispersed throughout the human genome [25] and, therefore, serve as excellent controls for the ZSCAN4 effect. No increase in acetylation was observed in the Alu elements following ZSCAN4 induction (Supplementary Figure S2D). Quantification of our dot blot data by ImageJ showed a significant 2.38 ± 0.18-fold increase in H3K14ac (*p* < 0.01) and a 1.67 ± 0.25-fold increase in H3K18ac (*p* < 0.01) (Suppl. Figure S2E) in the telomeric region. Conversely, the control Alu element sequence results indicated no significant change in H3K14 or H3K18ac in Alu elements (Supplementary Figure S2F), suggesting that telomeres are major targets of ZSCAN4-mediated histone H3 acetylation.

**Figure 4:**
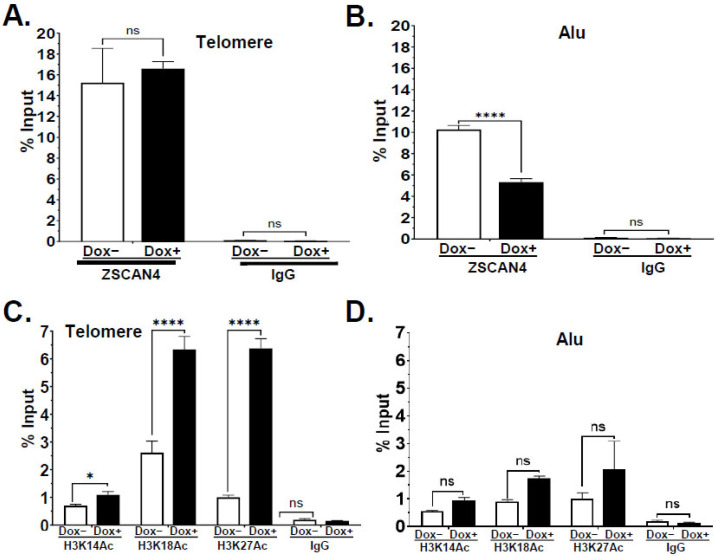
ZSCAN4 binds to telomeres, leading to increased histone H3 acetylation. The “% Input” values represent the enrichment of ZSCAN4, Alu, or histone acetylation on a specific region from each sample: without (Dox−) or with ZSCAN4 induction (Dox+): (**A**) ZSCAN4 interaction with the telomeres was assessed by ZSCAN4-ChIP, followed by telomere qPCR and (**B**) Alu qPCR as control. (**C**) The effect of ZSCAN4 induction for 48 h on telomere-histone H3 acetylation was assessed by ChIP using H3K14ac or H3K18ac antibodies, followed by telomere qPCR. (**D**) Alu repeats were used as controls. Data are shown as mean ± S.E.M. * *p* < 0.05. **** *p* < 0.0001. ns—not significant.

The effect of ZSCAN4 induction on telomere-histone acetylation was further validated through a ZSCAN4-ChIP assay, followed by telomere qPCR. First, we performed a ZSCAN4 ChIP-seq in tet-ZSCAN4 cells. Consistent with the Southern blot and telomere Q-FISH results, the data showed a >1.6-fold increase in the average telomere length after 48 h of ZSCAN4 induction (Dox+) compared to the controls (Supplementary S3C). Interestingly, ChIP-qPCR data showed no significant increase in ZSCAN4 binding to the telomere after Dox induction when calibrated to % input (Figure 4A). This suggests that ZSCAN4 binds the elongated telomeres in a distribution comparable to the controls. Conversely, our data showed a 2-fold decrease in the association of ZSCAN4 with the Alu elements after ZSCAN4 induction (*p* < 0.001) (Figure 4B).

Then, we performed ChIP assays following ZSCAN4 induction using H3K14ac or H3K18ac antibodies, followed by qPCR analyses, using normal rabbit IgG as a negative control. Data were first calculated and compared to the corresponding input of each sample. Next, the effect of 48 h of ZSCAN4 induction on histone acetylation was calculated and compared to untreated control cells (Dox−). In agreement with the ChIP dot blot, ChIP-qPCR data indicated a significant increase in H3K14ac, H3K18ac, and H3K27ac in the telomere region (Figure 4C). Inversely, ChIP-qPCR of control Alu element sequences showed non-significant changes in histone H3 acetylation (Figure 4D). Overall, these data suggest that ZSCAN4 is associated with and facilitates histone H3 acetylation at the telomere chromatin.

### 2.5. ZSCAN4 Knockout Leads to Telomere Shortening and a Decrease in H3K14ac

**Figure 5:**
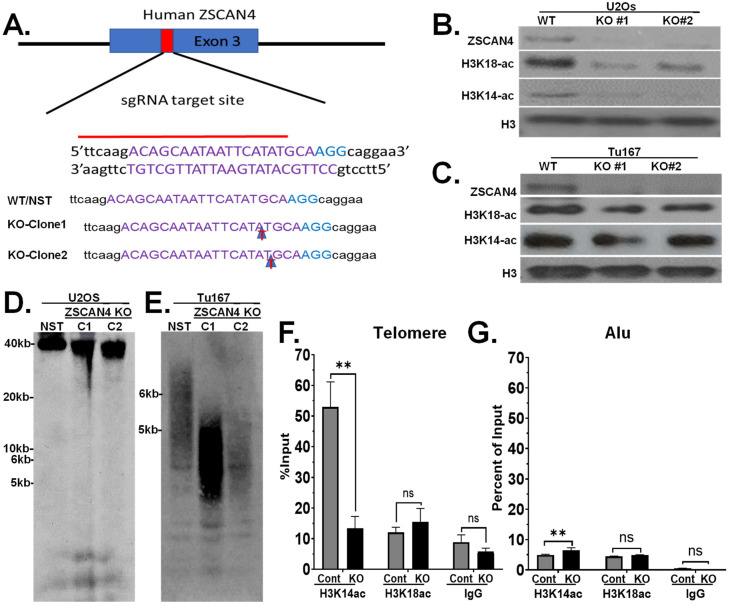
ZSCAN4 CRISPR/Cas9 knockout leads to telomere shortening and reduced histone H3 acetylation. (**A**) Sequencing data showing the wild-type (WT) sequence of ZSCAN4 and two ZSCAN4 CRISPR knockout clones (U2OS) with +1 nucleotide insertion, causing frameshift, stop codon, and loss of the ZSCAN4 protein. (**B**) Western blot analyses further confirm ZSCAN4 knockout and show a significant reduction of histone H3K14 and H3K18 acetylation in two knockout clones (C1 and C2) of U2O-S, and (**C**) two knockout clones of Tu167 cells and controls expressing a vector containing CAS9 without a specific guide RNA were used as controls. (**D**) Telomere Southern blot analyses in two ZSCAN4 knockout clones of U2OS cells and (**E**) Tu167 cells showed significant telomere shortening compared to the non-specific target control (NST). A biotinylated DNA ladder was used to assess telomere length. (**F**) Telomere and (**G**) Alu H3K14ac and H3K18 ChIP, followed by qPCR in ZSCAN4 knockout (Tu167) compared to control cells (Cas9 with nonspecific target). Data are shown as the mean ± S.E.M. ** *p* < 0.01. ns—not significant.

Next, to further define the role of ZSCAN4 in chromatin remodeling, using CRISPR/Cas9 technology, we generated ZSCAN4 knockout (KO) in multiple cell lines. To generate ZSCAN4 CRISPR knockout lines, cells (Tu167 and U2OS) were nucleofected with Cas 9 RNP and a ZSCAN4-specific sgRNA. We targeted exon 3, the first coding exon of human ZSCAN4. The U2OS and Tu167 ZSCAN4 KO clones were validated using Sanger Sequencing (Figure 5A). We then generated at least two knockout clones per cell line. Our immunoblot analyses further validated the efficient ZSCAN4 KO in U2OS (Figure 5B) and Tu167 (Figure 5C) cells in two independent clones.

Southern blot analyses were used to measure the effect of ZSCAN4 knockout on telomeres. Clones of wild-type (WT) with Cas9 and nonspecific targets were used as controls. Our results indicated that ZSCAN4 knockout in two clones of U2OS (Figure 5D) and Tu167 (Figure 5E) cells led to a major reduction in telomere length compared to the non-specific target control (NST).

Consistent with the increase in H3 acetylation after ZSCAN4 induction, we found that ZSCAN4 knockout leads to a significant reduction in acetylation of H3K14ac at the telomeres, but not H3K18ac, compared to control cells with Cas9 with nonspecific target (NST) (Figure 5F). A decrease in H3 acetylation marks was not observed in Alu repeats, which showed a minor increase in H3K14ac (Figure 5G). These data were validated using two independent knockout clones (Tu167; n = 3 per group). These results further suggest that ZSCAN4 promotes histone H3 acetylation specifically at telomeres to allow telomere extension mechanisms to act upon the telomeres.

### 2.6. Telomere Factors Are Maintained during ZSCAN4 Induction

Our Western blot analyses indicated no significant detectable changes in the global abundance of shelterin complex components TRF1, TRF2, and POT1 (Supplementary Figure S3A) up to 72 h after ZSCAN4 induction (Tu167). Therefore, to further study the effect of ZSCAN4 specifically on telomeres, we performed a ChIP assay with telomeric repeat-binding factor 1 (TRF1) as a method to specifically pull down telomere chromatin. Normal rabbit IgG was used as a negative control. Tu167 tet-ZSCAN4 cells were either induced with Dox (Dox+) for 48 h or maintained untreated (Dox−). Telomere-specific primers were used to detect the abundance of TRF1 in the telomere chromatin. RPLP0, Alu, and Intergenic primers were used as controls for global chromatin targets. Our TRF1 ChIP assay showed efficient pull-down of telomere chromatin (Supplementary Figure S3B) and confirmed telomere extension (Supplementary Figure S3C). These results suggest that ZSCAN4-induced histone H3 acetylation is not accompanied by telomere deprotection.

## 3. Discussion

Factors that control replicative lifespan are of great interest as cancer therapeutic targets. This is because, in contrast to unique mutations specific to each cancer type or tumor, the ability to maintain telomere length and replicative lifespan is a universal hallmark of cancer. Therefore, understanding the underlying mechanisms may lead to the development of more global cancer-specific therapies. Cancer and pluripotent stem cells reactivate survival and embryonic programs to maintain their replicative lifespan. The key, and necessary, steps in genome reprogramming of embryonic and cancer cells are chromatin remodeling and telomere elongation. Not surprisingly, stem cells and cancer cells are characterized by more open and permissive chromatin signatures and specific active histone marks [35,36,37,38,39].

Pluripotent stem cells, which are telomerase-positive, maintain the telomeres and their unlimited replicative capacity through activation of *Zscan4*, independent of telomerase [8,12,18,40].The telomere DNA is normally maintained in a tightly closed chromatin state (heterochromatin), further protected by the shelterin complex, making it inaccessible to modifying enzymes [15,41,42]. Accordingly, their DNA is methylated and wrapped around histone octamers, which are marked by high methylation (hypermethylated) and low histone acetylation (hypoacetylated) [7,26]. This prevents telomere maintenance mechanisms, such as telomerase [43], or the alternative lengthening of telomeres (ALT) [44] from repairing and extending the telomeres during DNA replication. Therefore, epigenetic modifications play an important role in telomere stability and length maintenance. Although many factors involved in telomere chromatin condensation have been identified [7], the mechanisms contributing to the switch to more accessible chromatin are not well understood. Yet, inhibition of deacetylases such as HDAC1 was shown to lead to an increase in histone acetylation and telomere extension [26].

Mouse embryonic stem cells (mESC) transiently express *mZscan4* [8], which correlates with global chromatin de-condensation [12,15,20] and telomere extension [8,12,15]. *mZscan4* has also been shown to be indirectly involved in global DNA demethylation through interaction with ubiquitin ligases, which sequester DNA methyltransferases [12,45,46]. We recently reported that the human ZSCAN4 is also transiently expressed, thereby increasing the frequency of cancer stem cells, in HNSCC [24]. However, the mechanism by which human ZSCAN4 alters telomere-chromatin remodeling remains to be characterized.

Histone tail acetylation reduces their positive charge, and as a result, their affinity to the negatively charged DNA. Histone acetylation, therefore, makes the chromatin more accessible. Our study reveals a novel role for human ZSCAN4 in facilitating histone H3 acetylation at the telomeres and demonstrates its importance for telomere maintenance in cancer cells. Our data show that human ZSCAN4 interacts with the telomeres (Figure 1 and Figure 4A,B), and its transient induction leads to telomere extension (Figure 2 and Figure 3). Remarkably, we show for the first time, that following its recruitment, ZSCAN4 leads to a significant increase in histone H3 acetylation at the telomeres at multiple lysine residues of histone H3 (H3K14, K18 and K27). Our results further show that ZSCAN4-mediated histone H3 acetylation is not accompanied by a reduction in the telomere shelterin complex (Supplementary Figure S3A). This is important, as the shelterin protects the telomere integrity.

Moreover, we show that CRISPR/Cas9 knockout of ZSCAN4 leads to significant telomere shortening in both Tu167 (telomerase-positive) and U2OS (telomerase-negative) cells (Figure 5) and a dramatic decrease in H3K14 acetylation, specifically at the telomeres. Our telomere ChIP data show a dramatic decrease in H3K14ac at the telomeres, but not in H3K18ac and K27ac. These data suggest that H3K14 acetylation plays a critical role in maintaining telomeres. Our data suggest that ZSCAN4 does not directly elongate telomeres; instead, it binds the telomeres and promotes functional histone H3K14 acetylation to facilitate telomere editing. Further research is necessary to identify the specific histone H3 acetyltransferase involved in these processes, in the absence or presence of ZSCAN4.

The expression of ZSCAN4 is not detectable in normal adult differentiated cells, making it an attractive target for cancer therapy. Our research suggests ZSCAN4 may represent a new link between telomere maintenance and the cancer stem cell phenotype. Understanding the mechanism by which ZSCAN4 affects telomere chromatin remodeling is important for the development of future therapeutic approaches targeting the replicative lifespan of cancer stem cells. Blocking ZSCAN4 alone, or in conjunction with additional chemotherapeutic drugs, may offer a new approach for cancer treatment with a wider therapeutic window and means to restore cancer replicative aging in novel therapeutics.

## 4. Materials and Methods

### 4.1. Cell Lines and Cell Culture

The head and neck squamous cell carcinoma (HNSCC) cell line (Tu167) was generated by Dr. Gary Clayman [47] and authenticated free from cross contamination [48]. U2OS and 293T cells were purchased from the ATCC. Other cell lines used (Hela, 012SCC, SKBr3, SW480) were authenticated and confirmed as mycoplasma-free by the University of Maryland translational core facility. Cells were cultured in complete DMEM medium (Gibco / Thermo Fisher Scientific, NY, USA) supplemented with 10% fetal bovine serum (FBS, tet free, US origin) (CPS Serum), 2 mM GlutaMAX, penicillin (100 U/mL) and streptomycin (100 μg/mL).

### 4.2. Generation of Doxycycline Inducible ZSCAN4 Cells

pZscan4-MP02 (doxycycline-inducible tet-ZSCAN4) vectors encoding lentiviral vectors and viral particles were generated as previously described [24]. Briefly, lentiviral particles were generated in 293T packaging cells cultured at 60% confluence, and transfected overnight, using Effectene (QIAGEN, Germantown, MD, USA). The ratio of 2 µg pZscan4-MP02, 1.2 µg of pLenti17531 and 0.8 µg pCMV-VSVG vectors was used per transfection. The medium containing viral particles was collected, filtered through a 0.45 μm filter and stored at −80 °C. Cells were transduced and puromycin selected to generate tet-ZSCAN4 cell lines.

### 4.3. Generation of ZSCAN4 CRISPR/Cas9 Knockout Clones

ZSCAN4 CRISPR/Cas9 knockout was conducted by the University of Maryland CRISPR Services Core Facility at Dr. Tami J. Kingsbury’s laboratory. Wild-type Tu167 or U2OS cells were nucleofected with a Cas9 RNP (Amaxa 4D system) and sgRNA sequence (CAGCAATAATTCATATGCA) targeting the first coding exon of human ZSCAN4. Clones were isolated, propagated and confirmed by Sanger Sequencing. ZSCAN4 knockout was further validated by immunoblot analyses.

### 4.4. Telomere Quantitative Fluorescence In Situ Hybridization (Q-FISH)

Q-FISH was performed as we previously described [49]. Briefly, all cells were maintained in the indicated conditions. On the harvesting day, 0.1 µg/mL colcemid (Invitrogen) was added for 6 h. Cells were collected by Accutase, incubated with 0.075 M KCl, fixed in cold methanol/acetic acid (3:1) and stored at 4 °C. Metaphase spreads were prepared as previously described [50,51], and telomere FISH was performed using a red fluorescence-conjugated Telomere PNA probe (AF546-OO-CCCTAACCCTAACCCTAA) (IDT). Chromosomes were stained with 0.5 μg/mL DAPI. For quantification and fluorescence intensity of telomere size, metaphase spreads and telomeres were captured by Nikon CSU-W1 Spinning Disk Confocal microscope and analyzed by TFL-TELO [28].

### 4.5. Co-Immunohistochemistry with Telomere FISH

Co-FISH was performed as previously described [8]; briefly, high-quality metaphase spreads were prepared, and unmasking was performed in citrate buffer at 90 °C. Slides were dehydrated and incubated for 5 min at 87 °C with Alexa568-conjugated (red) DNA probe (TTAGGGTTAGGGTTAGGG/3AlexF568N/ (IDT, Coralville, IA, USA) and allowed to anneal at room temperature for an hour. Primary antibodies, mouse anti-ZSCAN4 (1:1000) (Origene, Rockville, MD, USA), were diluted in blocking solution and incubated at 4 °C overnight. Slides were washed and incubated for an hour at room temperature with secondary antibodies (diluted in blocking solution) Alexa 488 Donkey anti mouse (1:400) (Invitrogen). Nuclei were counterstained with DAPI. Cells were visualized by Zeiss LSM 710-confocal microscope [52]. For quantification, co-localization was calculated by Fiji/ImageJ 2.14.0 software [52,53,54] from at least 20 nuclei in 5 replicates, in 3 independent experiments.

### 4.6. Terminal Restriction Fragment (TRF) Length Southern Blot Analysis

To prepare genomic DNA, 3 × 10^6^ cells were lysed in SDS Buffer (0.5% SDS in 200 mM Tris (pH 8.1), 25 mM EDTA, 250 mM NaCl) and treated with RNAse A (40 ng/mL), followed by proteinase K (20 ng/mL). DNA was extracted with phenol:chloroform and precipitated with isopropanol. Terminal DNA fragments were generated from 5 μg of genomic DNA by restriction digestion with HinfI and RsaI, followed by electrophoresis in 0.5% agarose gel, transferred to a Hybond-N+ membrane (Amersham), and incubated at 80 °C for 2 h. Bands were detected by hybridization with a 3′ biotin-labeled probe (CCCTAACCCTAACCCTAA-bio) (IDT) and visualized on X-ray film using a Chemiluminescent Nucleic Acid Detection Kit (Pierce) according to the manufacturer protocol.

### 4.7. Quantitative Reverse Transcription Polymerase Chain Reaction (qRT-PCR)

RNA was isolated using Trizol according to the manufacturer protocol (ThermoFisher, NY, NY, USA), and 1 µg was reverse transcribed by Superscript III (Invitrogen) following the manufacturer’s protocol. For qPCR, 10 ng cDNA was used per well in triplicate using SYBR green (Roche, Wrightsville, GA) following the manufacturer’s protocol. Reactions were run on a QuantStudio3 Real-Time qPCR system (Fisher Scientific, NY, NY, USA). Fold induction was calculated by the relative quantification method. The following primers were used: ZSCAN4 forward 5′-ATCCACCTGCCTTAGTCCAC-3′, reverse 5′-TCGAAGAACTGTTCCAGCCA-3′; RPLP0 forward 5′-CAGCAAGTGGGAAGGTGTAATCC-3′ and reverse 5′-CCCATTCTATCATCAACGGGTACAA-3′; hTERT Forward 5′-GGAGCAGTTGCAAAGCATTG-3′, reverse 5′-TCCCACGACGTATACATGTT-3′; hTERC forward 5′-TTTGTCTAACCCTAACTGAGAAGG-3′ and reverse 5′-CTCTAGAATGAACGGTGGAAGG-3′.

### 4.8. Quantitative Polymerase Chain Reaction (qPCR) Analyses

qPCRs were performed using an Applied Biosystems QuantStudio3 Real-Time qPCR system (Fisher Scientific). Primers used: hTELO-F: GGTTTTTGAGGGTGAGGGTGAG-GGTGAGGGTGAGGGT, hTELO-R: TCCCGACTATCCCTATCCCTATCCCTATCCCT-ATCCCTA; RPLP0 primers: hRPLP0-F: CAG CAAGTGGGAAGGTGTAATCC, hRPLP0-R: CCCATTCTATCATCAACGGGTACAA; Alu primers: Alu-F: GTCAGGAGATCGAGA CCATCCC, Alu-R: TCCTGCCTCAGCCTCCCAAG. A standard DNA calibration curve was also analyzed by serial dilution of genomic DNA from 50 ng to 1.25 ng. The results were calculated as a percentage of corresponding input samples. Data are visualized in charts as mean ± S.E.M. from at least 3 independent experiments. Data were analyzed by two-way ANOVA, corrected for multiple post hoc comparisons using the Bonferroni–Dunn method.

### 4.9. Chromatin Immunoprecipitation (ChIP)-qPCR

To study the association of ZSCAN4 with the telomeres, tet-FLAG-ZSCAN4-Tu167 cells were grown to 50% confluency and incubated for 48 h with or without 1 µg/mL doxycycline. ChIP was performed using the Pierce Magnetic ChIP Kit (Thermo Scientific) according to the manufacturer’s instructions. Antibodies used for ChIP: Anti-ZSCAN4 (10 µg) (Origene); anti-H3K14ac, H3K18ac, or H3K27ac (4 µg) (Cell signaling, Danvers, MA) and the provided anti-RNA Polymerase II (1 µg) and a Rabbit IgG (10 µg) were used as controls. DNA was purified using a PCR Clean Up Kit (Qiagen), and qPCR further performed.

### 4.10. ZSCAN4-ChIP Followed by Next Generation Sequencing Analyses

ZSCAN4 chromatin immunoprecipitation (ChIP) samples were taken for next-generation sequencing to study the effect of ZSCAN4 induction and the localization of ZSCAN4 at the telomeric sequences by using TelomereHunter software [31]. Briefly, the TelomereHunter application searches for telomere variant repeats (TVRs) of the type NNNGGG (and the reverse complement) in the intratelomeric reads, where ‘N’ can stand for A, C, G or T. Then, the software extracts 18 bp sequences on either side of the above TVRs and counts the different occurring combinations. Next, the number of intratelomeric reads are normalized by the number of comparable GC content (48–52%) reads and multiplied by 10, a unit we abbreviated as TRPM (telomeric reads per GC content-matched million reads). Lastly, the telomere content tumor/control log 2 ratio (log2 T/C) is computed. Statistical analyses and additional visualization were performed by GraphPad Prism 8.1.0 using two-way ANOVA corrected, followed by multiple post hoc comparisons tests.

### 4.11. Dot Blot Analyses

Purified DNA from Chromatin Immunoprecipitation (ChIP) samples was loaded to the Dot blot apparatus onto a Hybond-N positively charged nylon membrane by vacuum. To visualize the telomeric DNA, a 3′-biotinylated C-rich telomere probe (Telo-C) was used: CCCTAACCCTAACCCTAA. As control, we used a 3′-biotinylated mutated telomere repeats (Telo-Mut) probe: TTGGCGTTGGCGTTGGCG. For Alu repeat, a 3′-biotinylated Alu [25] probe (Alu) sequence: GGCCGGGCGCGGTGGCTCACGCCTGTAATCCCAGA was used. After an overnight hybridization, membranes were probed with streptavidin and detected by Chemiluminescent Nucleic Acid Detection Module Kit (ThermoFisher/Pierce) according to the manufacturer protocol. Dot blot quantification was analyzed by Fiji software [54], and data are shown as mean ± SEM. Statistical significance was calculated using GraphPad Prism 8.1.0 by two-way ANOVA and Bonferroni post hoc tests.

### 4.12. Telomerase Activity Measurement

All the cells in different passages were cultured in triplicate in complete medium and harvested after 2 days. Cell lysates were prepared from 10^6^ cells per sample. Telomerase activity was measured by real-time qPCR using a TRAPEZE Telomerase Detection Kit (Millipore, Chemicon, USA) according to the manufacturer’s instructions. Isogenic WT cells and telomerase-positive HeLa cells were used as positive controls. Other technical negative controls used were heat-inactivated extracts for each sample. Results are shown as mean ± S.E.M. in three biological replicates obtained from 3 independent experiments. Data were analyzed by two-way ANOVA.

### 4.13. Statistical Analyses

Results are shown as the mean ± S.E.M. of multiple independent experiments, with biological replicates. Detailed n values for each panel in the figures are stated in the corresponding figure legends. Student’s *t*-test and one-way or two-way ANOVAs followed by post hoc comparison tests (when appropriate) were used for the indicated statistical analyses. All statistical analyses were performed with STATISTICA 12 and GraphPad Prism 8.1.0 software. *p* values < 0.05 were considered statistically significant.

## Data Availability

All data generated or analyzed in the current study are presented in this manuscript of otherwise available from the corresponding author upon reasonable request. ChIP-Seq fastq data and bigwig files were deposited in GEO (GSE250470).

## Supplementary Materials

The supporting information can be downloaded at: https://www.mdpi.com/article/10.3390/ijms25073898/s1
