# Supplementary material for: Reprogramming Chromosome Ends by Functional Histone Acetylation
Source: Int J Mol Sci. 2024 Mar 31;25(7):3898. doi: 10.3390/ijms25073898 (PMC11011970; doi:10.3390/ijms25073898)
Supplement: Supplementary file 1 [file ijms-25-03898-s001.zip › ijms-2874928-supplementary.pdf]

Suppl. Figure S1

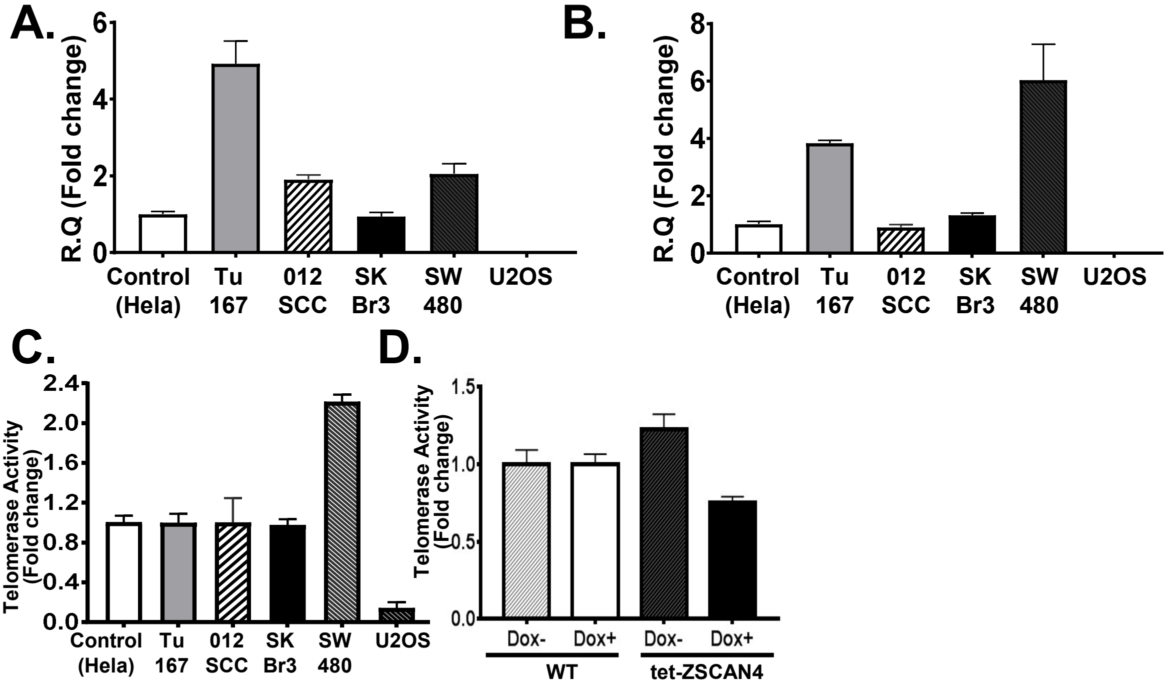

Supplementary Figure S1. ZSCAN4 does not significantly affect telomerase activity. Validation of telomerase status across multiple cell lines tested: qPCR data demonstrates expression levels of **A**. hTERT and **B**. hTERC in telomerase positive (Tu167, 012SCC, SKBr3, SW480) and telomerase negative (U2OS). Hela cells were used as positive control. **C.** Telomerase activity (TRAPeze RT) qPCR assay confirm the status of telomerase in our cell lines. Negative controls include heat treated samples, no Taq polymerase samples and no telomerase samples per each cell line. Positive control is sample provided by TRAPeze RT assay (Millipore) and Hela cells. **D**. ZSCAN4 induction does not lead to a significant increase in telomerase activity and a mild reduction is observed as shown by Telomerase TRAPeze RT real time qPCR. Controls: isogenic wild type (WT) Tu167 cells in untreated and Dox treatment conditions. Additional technical controls (not shown): telomerase positive HeLa cells. Negative controls used (not shown): heat inactivated extracts per each sample. Error bars indicate S.E.M.

Suppl. Figure S2

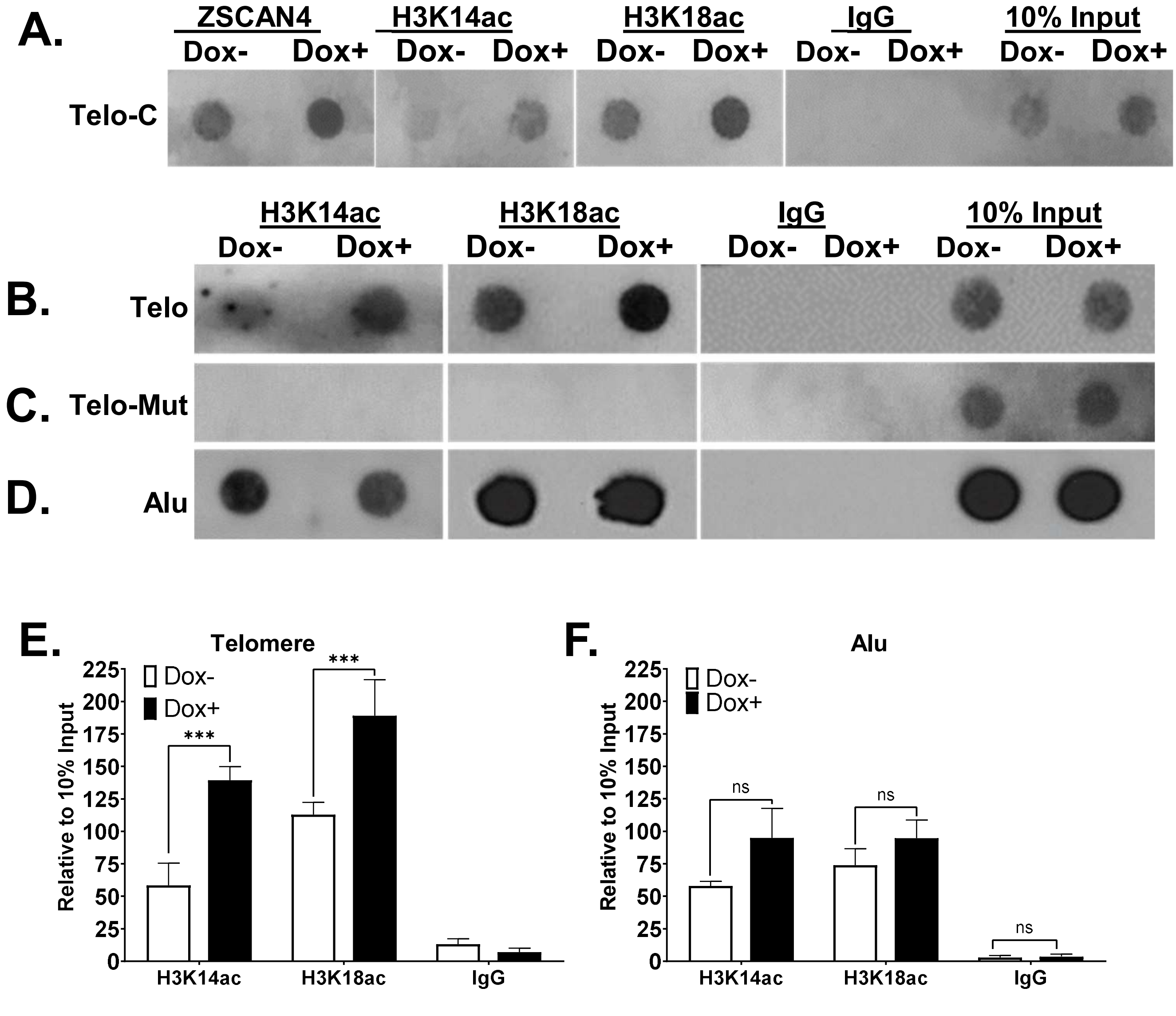

Supplementary Figure S2: **ZSCAN4 induction leads to increase in histone H3 acetylation at the telomeres**. Representative images of ChIP assays with the indicated histone H3 acetylation antibodies followed by dot blot analyses in Tu167 tet-ZSCAN4 cells treated with doxycycline (Dox+) or untreated (Dox-). **A.** Telomere dot blot analyses indicate an increase in telomere content. Therefore, samples were diluted to **B**. demonstrate an increase in telomere histone acetylation at H3K14ac and H3K18ac. **C.** Dot blot with TeloMut control probe show no H3 acetylation while **D**. Alu element control probe show no significant enrichment in histone H3 acetylation. 10% Input was used for loading controls. IgG were used as negative controls. **E**. Quantitative analyses of telomere dot blot relative to 10% input by ImageJ indicate a significant increase acetylation of H3K14ac and H3K18ac. **F.** Quantitative analyses of Alu dot blot by ImageJ indicate H3 acetylation were not significantly changed upon ZSCAN4 induction. All data shown as mean ± S.E.M from 4 independent experiments. Significance was calculated by two-way ANOVA followed by individual post hoc tests. *** $p < 0.0001$.

Suppl. Figure S3

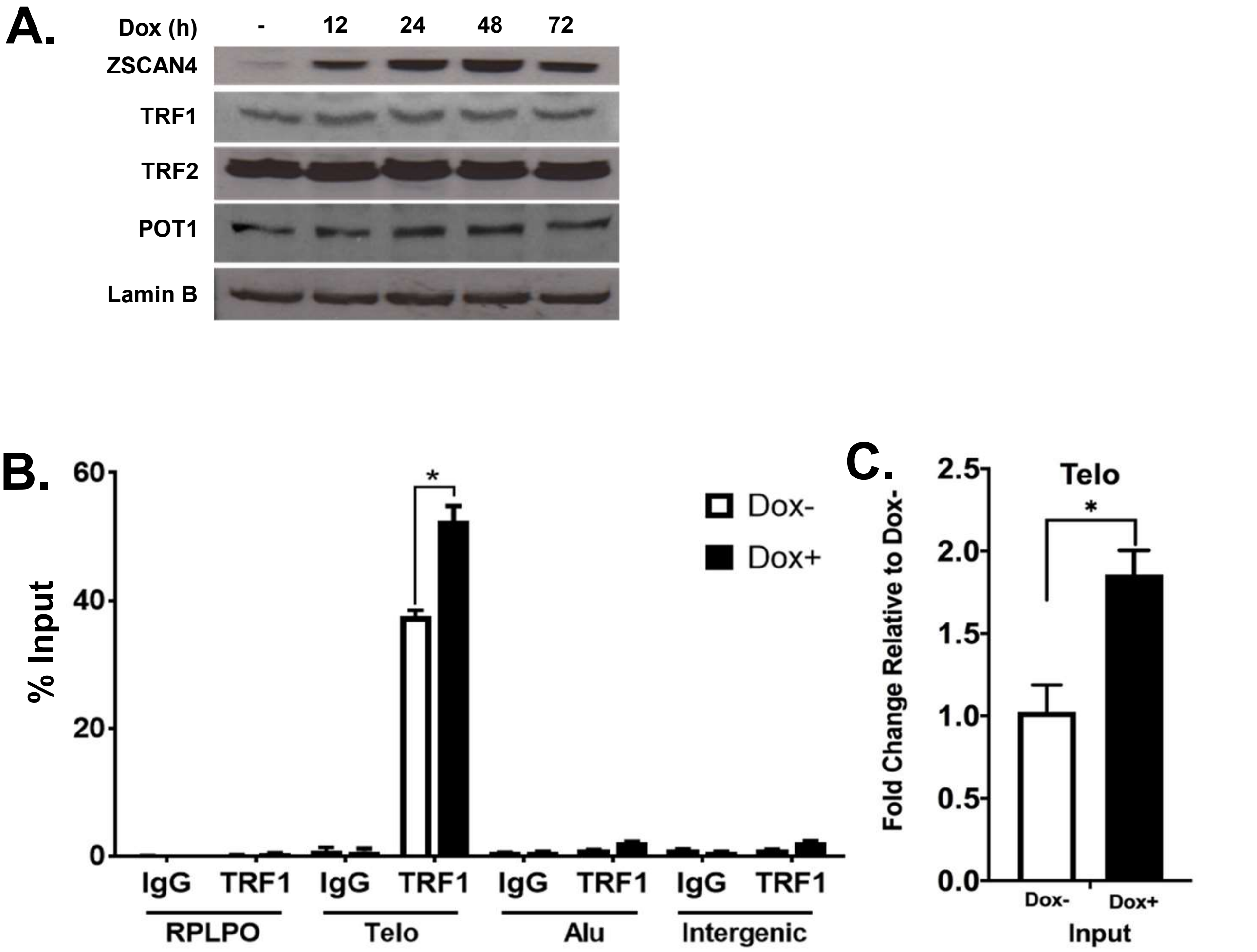

Supplementary Figure S3. ZSCAN4 chromatin modification is not accompanied by telomere deprotection or reduction in the shelterin complex. **A**. Immunoblot analyses show that ZSCAN4 induction does not affect shelterin complex component proteins. Control: Lamin-B (nucleus). **(A)** Quantitative qPCR analyses of ChIP samples using TRF1 antibody analyzed by RPLPO, Telo, Alu and Intergenic primers. **(B)** Quantitative qPCR data study of telomere DNA from input samples comparing the relative fold change between Dox+ and Dox-samples. Data shown as mean ± S.E.M. The statistical significance between the two groups was determined by separate t-tests * $p < 0.05$. * $p < 0.05$.
